# RP-182 alleviated obstruction-induced renal fibrosis by reprogramming CD206^+^ macrophages

**DOI:** 10.3389/fphar.2026.1739457

**Published:** 2026-01-30

**Authors:** Hualin Cao, Xiaoye Chen, Ruyue Jin, Yunjie Yang, Yuandong Tao, Pin Li, Yangyang Wu, Guilong Chen, Jiawen Zhao, Jianhua Wen, Yutong Zhao, Liwei Wei, Zhengshu Wei, Dingjin Lu, Yuekang Chen, Dehong Liu, Huixia Zhou, Jiwen Cheng

**Affiliations:** 1 Department of Urology, The First Affiliated Hospital of Guangxi Medical University, Nanning, China; 2 Nanxishan Hospital (The Second People’s Hospital) of Guangxi Zhuang Autonomous Region, Guilin, China; 3 Department of Pediatric Urology, Chinese PLA General Hospital, Beijing, China; 4 The Second School of Clinical Medicine, Southern Medical University, Guangzhou, China; 5 Department of Pediatric Surgery, The Sixth Affiliated Hospital, School of Medicine, South China University of Technology, Foshan, China; 6 Department of Pediatric Surgery, Ruijin Hospital Affiliated to Shanghai Jiaotong University School of Medicine, Shanghai, China

**Keywords:** CD206+ macrophages, obstructive nephropathy, renal fibrosis, RP-182, UUO

## Abstract

**Introduction:**

Obstructive nephropathy is a major cause of chronic kidney disease (CKD), characterized by progressive renal fibrosis with limited treatment options. CD206^+^ macrophages have emerged as key drivers of fibrogenesis, yet targeted strategies against this subset remain undeveloped.

**Methods:**

Using human ureteropelvic junction obstruction (UPJO) tissues and a murine unilateral ureteral obstruction (UUO) model, we assessed the accumulation of CD206^+^ macrophages and the progression of fibrosis. The therapeutic peptide RP-182, which selectively targets CD206, was administered daily to UUO mice. Histological, molecular, and flow cytometric analyses were performed to evaluate renal injury, fibrosis, inflammation, and macrophage polarization. *In vitro* studies using bone marrow-derived macrophages elucidated the mechanisms underlying the action of RP-182.

**Results:**

CD206^+^ macrophages were significantly enriched in human UPJO kidneys and UUO mice, correlating with fibrosis severity. RP-182 treatment attenuated collagen deposition, α- SMA expression, tubular damage, and inflammatory cell infiltration in UUO kidneys. *In vitro*, RP-182 selectively inhibited IL-4/IL-13-induced M2 polarization and suppressed TGF- β-triggered macrophage-to-myofibroblast transition (MMT) in M2 macrophages, while sparing M1 responses. Mechanistically, RP-182 downregulated β-catenin signaling, a pathway crucial for M2 programming and MMT.

**Discussion:**

Our findings demonstrate that RP-182 alleviates obstructive renal fibrosis by specifically targeting CD206^+^ macrophages, inhibiting their M2 polarization and MMT via β-catenin suppression. This work highlights RP-182 as a novel macrophage- directed therapeutic candidate for progressive kidney fibrosis.

## 1 Introduction

Renal fibrosis, characterized by the excessive deposition of extracellular matrix (ECM), is a common pathological hallmark of chronic kidney disease (CKD) that leads to progressive scarring and irreversible loss of kidney function (Huang et al., 2023). It arises from diverse etiologies, including diabetes, hypertension, glomerulonephritis, and prolonged obstruction. Furthermore, environmental and metabolic factors, such as dietary advanced lipoxidation end products, can also initiate renal impairment through sustained inflammatory responses (Wang et al., 2025). These triggers converge on persistent inflammation and maladaptive repair processes that drive fibrogenesis (Norregaard et al., 2023). Epidemiologically, CKD affects over 800 million people globally, with renal fibrosis contributing significantly to its progression and the growing burden of end-stage renal disease (ESRD) (Kalantar-Zadeh et al., 2021; Wang et al., 2023). The devastating consequences of renal fibrosis include declining glomerular filtration rate, systemic complications such as cardiovascular disease, and ultimately the requirement for dialysis or kidney transplantation (Kalantar-Zadeh et al., 2021). Despite its clinical significance, no effective anti-fibrotic therapies are currently available (Zhao et al., 2020), highlighting an urgent need for novel therapeutic strategies targeting key players in fibrogenesis, such as macrophages.

Macrophages are central mediators of immune responses in kidney injury and fibrosis, exhibiting remarkable plasticity that allows them to adopt either pro-inflammatory or profibrotic phenotypes (Tang et al., 2019; Wang et al., 2021). Following persistent injury, macrophages accumulate extensively in the renal interstitium and dynamically participate in all stages of disease progression, from initial inflammation to extracellular matrix deposition and tissue scarring (Huen and Cantley, 2017). Classically activated M1 macrophages contribute to early tissue damage through the release of inflammatory cytokines such as TNF-α and IL-1β (Meng et al., 2022). However, as the injury persists, a shift toward alternatively activated M2 macrophages occurs. These M2 macrophages, especially those expressing CD206 (the macrophage mannose receptor), play a critical role in driving fibrogenesis (Wu et al., 2020). They secrete a plethora of profibrotic factors, including TGF-β1, PDGF, and IL-10, which directly activate resident fibroblasts and promote the transformation of epithelial cells into myofibroblasts via epithelial-mesenchymal transition (EMT) (Wang et al., 2021). Furthermore, they contribute to renal fibrosis through macrophage-myofibroblast transition (MMT) (Wei et al., 2022). Besides, macrophages lead to immune suppression and matrix remodeling by producing enzymes such as arginase-1 and matrix metalloproteinases (Wang et al., 2021). Recent research has underscored the crucial role of CD206-expressing M2 macrophages in promoting organ fibrosis, particularly in the kidney (Tang et al., 2019). Both clinical studies and murine models consistently demonstrate elevated CD206 expression correlating with the severity of fibrosis (Toki et al., 2014; Hou et al., 2018; Di et al., 2025). Genetic and pharmacological investigations directly implicate this subset in disease progression. In renal fibrosis models, targeted elimination of CD206+ macrophages or inhibition of their recruitment has been shown to reduce collagen deposition and myofibroblast activation (Kuppe et al., 2021; Ouyang et al., 2024). Moreover, general depletion of macrophages has consistently been found to impede the onset and advancement of renal fibrosis. For example, systemic administration of clodronate liposomes for macrophage depletion significantly mitigated renal injury and fibrosis in the unilateral ureteral obstruction (UUO) model (Duffield et al., 2005). Hence, targeting CD206+ macrophages has emerged as a promising therapeutic avenue for alleviating renal fibrosis, with potential strategies including cell depletion or phenotype reprogramming.

RP-182 is a synthetic analog of host defense peptides (HDPs) that selectively induces a conformational switch of the mannose receptor CD206 expressed on macrophages (Jaynes et al., 2020). A study demonstrates that RP-182 mediates activation of CD206 in human and murine M2-like macrophages, eliciting a program of endocytosis, phagosome-lysosome formation, and autophagy and reprogramming M2-like tumor associated macrophages to an antitumor M1-like phenotype (Jaynes et al., 2020). However, its therapeutic potential and underlying mechanisms in renal fibrosis remain to be explored. Specifically, we seek to determine whether RP-182 ameliorates fibrosis by selectively suppressing M2 macrophage polarization and inhibiting the MMT process.

## 2 Materials and methods

### 2.1 Patient samples

Human kidney tissues were obtained from two distinct patient cohorts. Healthy control kidney tissues were collected from patients undergoing nephrectomy for nephroblastoma, with sampling performed from macroscopically and histologically unaffected regions. Obstructed kidney tissues were acquired from patients with late-presenting ureteropelvic junction obstruction (UPJO) who required surgical resection. All tissue procurement procedures were approved by the Institutional Ethics Committee of Chinese PLA General Hospital. Written informed consent was obtained from all participants or their legal guardians prior to tissue collection.

### 2.2 UUO model

Wild-type C57BL/6 mice were obtained from SPF Biotechnology Co., Ltd. (Beijing, China). All experimental animals were maintained under specific pathogen-free conditions with a controlled temperature (22 °C) and humidity (55%), following a 12-h light/dark cycle. The experimental protocols were approved by the Institutional Animal Care and Use Committee (IACUC) of the Seventh Medical Center of Chinese PLA General Hospital. To establish the UUO model, 8-week-old male mice were used. Briefly, after anesthetizing the mice with Avertin (Sigma, T48402), a midline abdominal incision was made and the left ureter was double ligated. Sham-operated control mice underwent identical surgical procedures without ureteral ligation. Kidney tissues were collected 14 days after the UUO surgery. The RP-182 peptide (TFA removed, Novopro, 318940) was administered via intraperitoneal injection daily, beginning on the first day of modeling, at a dosage of 20 mg/kg body weight. This dosage regimen was selected based on its established efficacy in modulating CD206^+^ macrophages in prior *in vivo* studies (Jaynes et al., 2020).

### 2.3 Histological analysis

The mouse kidneys were harvested and fixed in 4% paraformaldehyde and embedded with paraffin for analysis. The kidney sections were stained with hematoxylin and eosin (H&E) and Masson’s trichrome. Images were obtained using a Nano Zoomer Slide Scanner (Hamamatsu Photonics). Collagen-positive areas were quantified using ImageJ software. Blinded histopathological evaluation was conducted by two independent investigators using a semi-quantitative scoring protocol adapted from previous publications (Lu et al., 2025). The scoring criteria were as follows: 0 (no injury), 1 (1%–20% affected area), 2 (21%–50% affected area), 3 (51%–75% affected area), and 4 (>75% affected area).

### 2.4 Immunohistochemistry and immunofluorescence

The mouse kidneys were fixed in 4% paraformaldehyde and embedded with paraffin. The kidney sections were stained with the following primary antibodies: anti-α-SMA (Proteintech, 67735-1-Ig), anti-CD206 (Proteintech, 18704-1-AP), anti-CD68 (Proteintech, 66231-2-Ig), and anti-F4/80 (Abcam, ab6640). The sections were counterstained with 4,6-diamidino-2-phenylindole dihydrochloride (DAPI) before being mounted. Imaging was performed using a Nano Zoomer Slide Scanner (Hamamatsu Photonics).

### 2.5 Immunoblots

Kidney tissue or macrophage protein extracts were prepared according to standard protocols. The tissue lysates were separated by SDS-PAGE and transferred to polyvinylidene difluoride membranes (Millipore). The following primary antibodies were used: anti-KIM1 (R&D, AF1817), anti-collagen I (Abcam, ab260043), anti-α-SMA (Proteintech, 67735-1-Ig), anti-β-actin (Proteintech, 66009-1-Ig), anti-Arginase 1 (Proteintech, 16001-1-AP),and anti-β-Catenin (Proteintech, 51067-2-AP). Bolt images were obtained on a ChemiDoc Imaging System (Bio-Rad). The quantification was performed using ImageJ software.

### 2.6 Quantitative PCR (qPCR)

The kidney tissues or macrophages were homogenized and total RNA was extracted using an RNA Extraction Kit (Huaxingbio, HXR8075) according to the manufacturer’s instructions. Complementary DNA was generated using a Reverse Transcription Kit (Takara, RR037A). Quantitative PCR was conducted on the JLM QX400 Real-time PCR System (Sichuan Jielaimei Technology Co. Ltd., China). The expression of the target gene was normalized to the expression of the housekeeping gene, *Gapdh*. Relative gene expression was calculated using the standard 2^−ΔΔCT^ method. The qPCR primers were listed in Table 1.

**TABLE 1:** The qPCR primers.

Genes	Forward	Reverse
*Gapdh*	AGG​TCG​GTG​TGA​ACG​GAT​TTG	TGT​AGA​CCA​TGT​AGT​TGA​GGT​CA
*Col1a1*	CCT​CAG​GGT​ATT​GCT​GGA​CAA​C	CAG​AAG​GAC​CTT​GTT​TGC​CAG​G
*Acta2*	ACT​GCC​GAG​CGT​GAG​ATT​GT	TGA​TGC​TGT​TAT​AGG​TGG​TTT​CG
*Fn*	CCC​TAT​CTC​TGA​TAC​CGT​TGT​CC	TGC​CGC​AAC​TAC​TGT​GAT​TCG​G
*Il1b*	TGT​AAT​GAA​AGA​CGG​CAC​ACC	TCT​TCT​TTG​GGT​ATT​GCT​TGG
*Il6*	CTG​CAA​GTG​CAT​CAT​CGT​TGT​TC	CTG​CAA​GTG​CAT​CAT​CGT​TGT​TC
*Tnf*	TCCAGGCGGTGCCTATGT	CAC​CCC​GAA​GTT​CAG​TAG​ACA​GA
*Tgfb1*	TGA​TAC​GCC​TGA​GTG​GCT​GTC​T	TGA​TAC​GCC​TGA​GTG​GCT​GTC​T
*Arg1*	TCG​GGT​CAT​GTT​CAA​GTC​CAG​C	GCT​GAA​GGT​CTC​TTC​CAT​CAC​C
*Kim1*	CTA​TGT​TGG​CAT​CTG​CAT​CG	AAG​GCA​ACC​ACG​CTT​AGA​GA
*Ym1*	CCC​TAT​CTC​TGA​TAC​CGT​TGT​CC	TGC​CGC​AAC​TAC​TGT​GAT​TCG​G
*Fizz1*	CAA​GGA​ACT​TCT​TGC​CAA​TCC​AG	CCA​AGA​TCC​ACA​GGC​AAA​GCC​A
*Mcp1*	GCT​ACA​AGA​GGA​TCA​CCA​GCA​G	GTC​TGG​ACC​CAT​TCC​TTC​TTG​G
*Nos2*	GAG​ACA​GGG​AAG​TCT​GAA​GCA​C	CCA​GCA​GTA​GTT​GCT​CCT​CTT​C

### 2.7 Flow cytometric analysis

Kidney leukocytes were isolated as previously described (Tao et al., 2023). Mice were anesthetized with Avertin and the kidneys were collected. The kidneys were minced into pieces and digested using 0.05% Collagenase IV (Sigma, C5138) and 2 mM CaCl_2_ at 37 °C for 25 min. Then, the digested kidney tissues were filtered through a 100 μm nylon mesh, followed by centrifugation at 500 *g* for 5 min and incubation with an Fcγ receptor blocker (BioLegend, 101320) for 10 min. The following fluorescent antibodies (all from BioLegend) were used: CD45-BV421 (103134), CD11b-FITC (101206), Ly6G-APC/Cyanine7 (127624), Ly6C-PE (128008), F4/80-APC (123116), CD206-PE/Cyanine7 (141720), CD3-PE (100206), CD4-PE/Cyanine7 (116016), CD8a-APC/Cyanine7 (100713), NK1.1-FITC (156508), and CD20-APC (152107). Flow cytometry was performed using a FACSCanto II (BD Biosciences). The data were analyzed using FlowJo software 10.4.

### 2.8 Cell culture and treatment

Bone marrow-derived macrophages (BMDMs) were prepared as previously described.(Jia et al., 2025). Briefly, bone marrow cells were harvested from femurs and tibiae of 6-week-old male mice. Cells were cultured in DMEM supplemented with 10% fetal bovine serum and M-CSF (Peprotech, 315-02, 50 ng/mL) for macrophage differentiation for 5 days with medium renewal on day 3. On day 5, BMDMs were polarized to M1 or M2 phenotype through 48-h stimulation with recombinant murine IL-4 (Peprotech, 214-14, 10 ng/mL) and recombinant murine IL-13 (Peprotech, 213-13, 10 ng/mL) or Lipopolysaccharide (Sigma, L2637, 100 ng/mL), respectively. To induce macrophage-to-myofibroblast transition (MMT), recombinant TGF-β (MCE, HY-P7118, 10 ng/mL) was used. In some experiments, BMDMs were stimulated with RP-182 peptide (TFA removed, Novopro, 318940) at concentrations of 20 or 40 μM, and with XAV-939 (10 mM, Selleck, United States) at a concentration of 10 μM.

### 2.9 Bulk RNA sequencing

Bulk RNA sequencing was performed on BMDMs treated with IL-4/13 + PBS or IL-4/13 + 40 μM RP-182 (Novogene, Beijing, China). Total RNA was extracted, and libraries were prepared and sequenced on an Illumina platform. Bioinformatic analysis included quality control, read alignment, differential expression analysis with DESeq2, and functional enrichment.

### 2.10 Statistical analysis

Statistical analyses were performed with GraphPad Prism (Version 10.1). The data are presented as mean ± standard error of the mean (SEM), with sample sizes explicitly stated in figure legends. Two-tailed Student’s t-test was used for comparisons between two groups with equal variance. One-way ANOVA was used to analyze three or more comparisons with equal variance. A p-value less than 0.05 was considered significant.

## 3 Results

### 3.1 Chronic ureteral obstruction resulted in significant renal fibrosis and marked accumulation of CD206^+^ macrophages in UPJO patients

**FIGURE 1:**
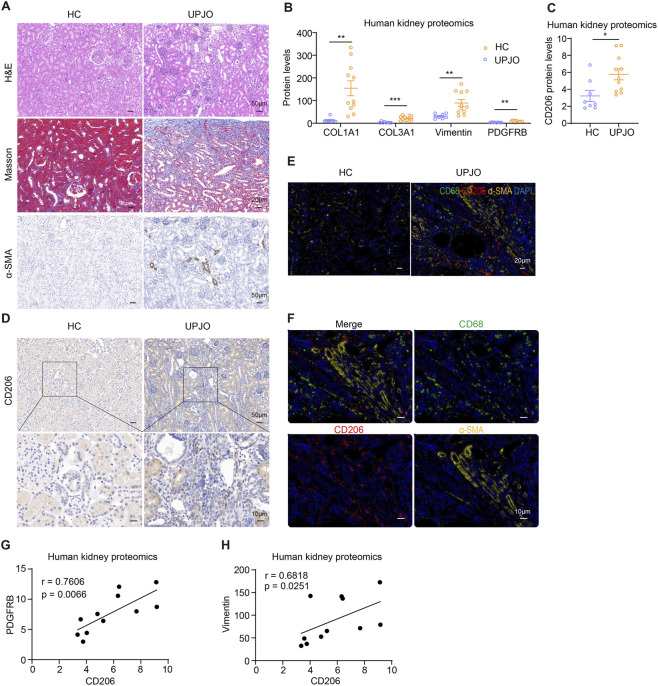
Chronic ureteral obstruction led to renal fibrosis and CD206^+^ macrophage accumulation in UPJO patients. **(A)** Representative kidney images of H&E, Masson, and immunohistochemistry staining for α-SMA in healthy control (HC) and UPJO patients. Scale bars, upper 50 μm, middle 20 μm, and lower 50 μm. The levels of fibrosis-associated proteins **(B)** and CD206 **(C)** in HC and UPJO patients. The data were from kidney proteomics. **(D)** Representative kidney images of immunohistochemistry staining for CD206 in HC and UPJO patients. Scale bars, upper 50 μm, lower 10 μm. **(E,F)** Representative kidney images of immunofluorescent staining for CD68, CD206, and α-SMA in HC and UPJO patients. Scale bars, **(E)** 20 μm, **(F)** 10 μm. **(G,H)** Pearson correlation analysis between the expression levels of CD206 and PDGFRB and Vimentin. The results represent mean ± SEM. *p < 0.05, **p < 0.01, ***p < 0.001.

We collected healthy renal tissues from patients undergoing nephrectomy for nephroblastoma and obstructive kidney specimens from UPJO patients. Histopathological analysis revealed that chronic obstruction led to extensive architectural disruption of renal tissues along with prominent leukocyte infiltration (Figure 1A). Masson’s trichrome staining further confirmed substantial collagen accumulation in the obstructed kidneys, indicative of progressive fibrosis (Figure 1A). Additionally, there was a significant increase in α-smooth muscle actin (α-SMA)-positive cells in UPJO tissues compared to healthy controls, suggesting enhanced activation of myofibroblasts (Figure 1A).

Consistent with these morphological changes, proteomic profiling of kidney tissues from our previous study showed pronounced upregulation of well-established fibrosis-related markers in the obstructed group, including COL1A1, COL3A1, Vimentin, and PDGFB (Figure 1B). Notably, CD206 expression was also significantly elevated in UPJO kidneys according to proteomic data (Figure 1C). Subsequent immunohistochemical staining confirmed the abundant infiltration of CD206^+^ cells within the interstitial compartments of obstructed kidneys, which was minimal in controls (Figure 1D). Double immunostaining experiments demonstrated frequent co-localization of CD206 with the pan-macrophage marker CD68, identifying these cells as macrophages (Figure 1E). Furthermore, CD206^+^ macrophages were often localized in close proximity to α-SMA-positive regions, implying a potential interaction with activated myofibroblasts (Figure 1F). Corroborating these spatial associations, proteomic correlation analysis revealed positive correlations between CD206 expression and fibrosis markers, such as PDGFB and Vimentin (Figures 1G,H). Collectively, these findings indicate that chronic ureteral obstruction promotes renal injury and fibrotic remodeling, accompanied by the accumulation of CD206^+^ macrophages, suggesting their contributory role in obstructive nephropathy.

### 3.2 Uniliteral ureteral obstruction caused renal fibrosis and inflammation characterized by CD206^+^ macrophage infiltration in mice

**FIGURE 2:**
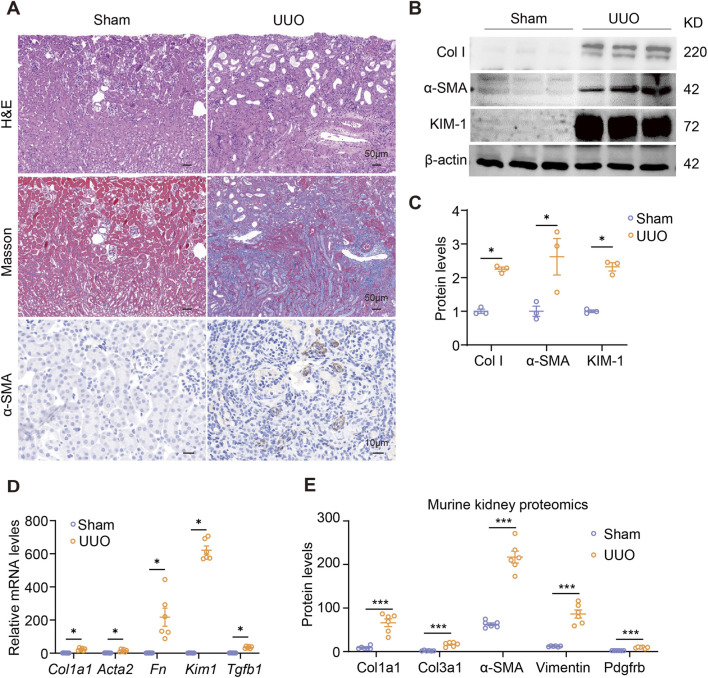
Characterization of UUO-induced renal fibrosis in mice. **(A)** Representative kidney images of H&E, Masson, and immunohistochemistry staining for α-SMA in sham and UUO mice. Scale bars, upper 50 μm, middle 50 μm, and lower 20 μm. Immunoblots **(B)** and quantification **(C)** of collagen I, α-SMA, and KIM-1 expression in sham and UUO kidneys. **(D)** qPCR analysis for *Col1a1*, *Acta2*, *Fn*, *Kim1*, and *Tgfb1* expression in sham and UUO kidneys. **(E)** The levels of fibrosis-associated proteins in sham and UUO mice. The data were from kidney proteomics. The results represent mean ± SEM. *p < 0.05, ***p < 0.001.

To model human obstructive nephropathy, we established a murine model of unilateral ureteral obstruction (UUO). Consistent with the pathological features observed in human UPJO tissues, UUO kidneys exhibited pronounced tubular dilation, substantial tissue injury, and widespread interstitial fibrosis. This was supported by significant collagen deposition, along with elevated expression of collagen I, α-SMA, and kidney injury molecule 1 (KIM-1) (Figures 2A–C). Transcript analysis and proteomic data further confirmed the pronounced upregulation of multiple fibrotic proteins in obstructed kidneys, including Col1a1, Col3a1, α-SMA, fibronectin (FN), TGF-β, Vimentin, and PDGFRB (Figures 2D,E), thereby validating the UUO model as a reliable representation of human obstructive nephropathy.

**FIGURE 3:**
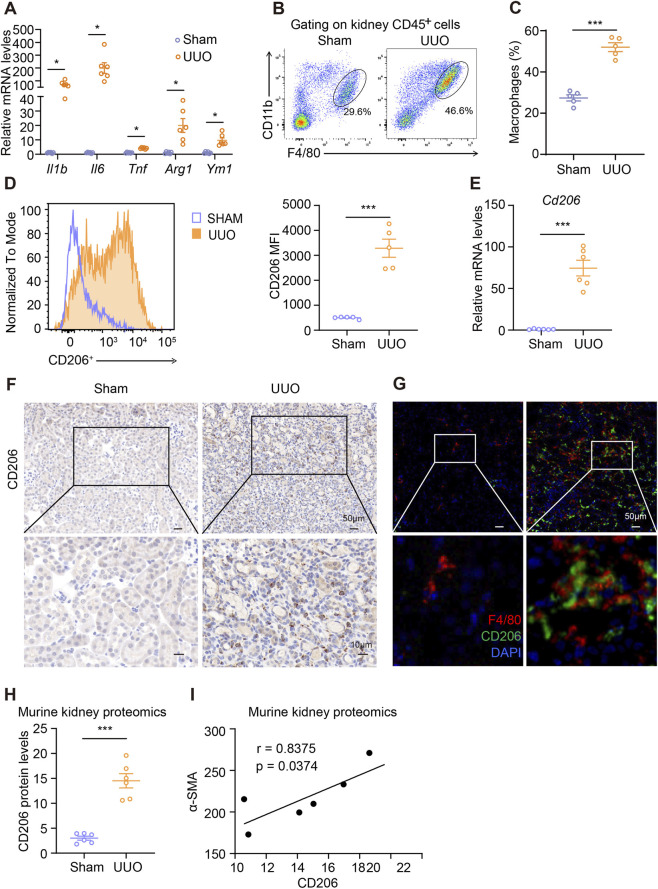
CD206+ macrophages were accumulated in UUO kidneys. **(A)** qPCR analysis for *Il1b*, *Il6*, *Tnf*, *Arg1*, and *Ym1* expression in sham and UUO kidneys. **(B,C)** Flow cytometry analysis of macrophages in sham and UUO kidneys. **(D)** Flow cytometry analysis of the expression of macrophage CD206 in sham and UUO kidneys. **(E)** qPCR analysis for *Cd206* expression in sham and UUO kidneys. **(F)** Representative kidney images of immunohistochemistry staining for CD206 in sham and UUO kidneys. Scale bars, upper 50 μm, lower 10 μm. **(G)** Representative kidney images of immunofluorescent staining for F4/80 and CD206 in sham and UUO kidneys. Scale bars, 50 μm. **(H)** The protein levels of CD206 in sham and UUO kidneys. The data were from kidney proteomics. **(I)** Pearson correlation analysis between the expression levels of CD206 and α-SMA. The results represent mean ± SEM. *p < 0.05, **p < 0.01, ***p < 0.001.

In addition to fibrotic changes, UUO kidneys displayed a robust inflammatory response. Transcript levels of key inflammatory cytokines, including *Il1b*, *Il6*, and *Tnf*, were significantly elevated compared to sham-operated controls (Figure 3A). We also detected increased expression of classic M2 macrophage markers *Arg1* and *Ym1*, suggesting a shift toward a profibrotic macrophage phenotype (Figure 3A). Flow cytometric analysis confirmed enhanced macrophage infiltration in UUO kidneys (Figures 3B,C), and notably revealed a specific upregulation of CD206 expression on renal macrophages following obstruction (Figure 3D). Moreover, CD206 transcript levels were significantly increased in UUO kidneys (Figure 3E), and histological examination showed substantial accumulation of CD206^+^ cells within the interstitial compartment compared to sham controls (Figure 3F). These CD206^+^ cells were further identified as macrophages via co-staining with the macrophage marker F4/80 (Figure 3G). Proteomic data also indicated a substantial elevation in CD206 protein levels in obstructed kidneys (Figure 3H). Taken together, these data demonstrate significant recruitment of CD206^+^ macrophages in the context of UUO-induced renal fibrosis. Importantly, a positive correlation was observed between α-SMA and CD206 protein levels (Figure 3I), supporting a potential role for CD206^+^ macrophages in promoting renal fibrogenesis.

### 3.3 Targeting CD206 by RP-182 peptide inhibited UUO-induced renal fibrosis and inflammation

**FIGURE 4:**
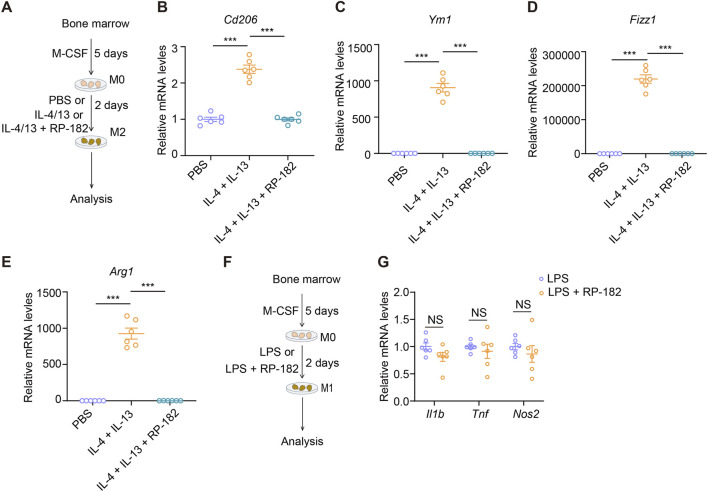
RP-182 peptide inhibited UUO-induced renal fibrosis. **(A)** The schematic of the experimental design. Representative kidney images and quantification of H&E **(B,C)** and Masson **(D,E)** staining in PBS- and RP-182-treated UUO mice. Scale bars, **(B)** upper 50 μm and lower 10 μm, **(D)** upper 50 μm and lower 10 μm. **(F)** Immunoblots and **(G)** quantification of renal expression of collagen I, α-SMA, and KIM-1 in PBS- and RP-182-treated UUO mice. The results represent mean ± SEM. *p < 0.05, **p < 0.01.

To evaluate the therapeutic potential of targeting CD206^+^ macrophages in renal fibrosis, we administered the CD206-binding peptide RP-182 to UUO mice (Figure 4A). Histopathological examination revealed that RP-182 treatment markedly attenuated tubular injury and reduced immune cell infiltration compared to PBS-treated UUO mice (Figures 4B,C). Consistent with these observations, Masson’s trichrome staining indicated a significant reduction in collagen deposition in RP-182-treated animals (Figures 4D,E). Furthermore, the expression of the fibrosis markers collagen I and α-SMA, as well as the renal injury marker KIM-1, was substantially downregulated following RP-182 intervention (Figures 4F,G). Together, these findings demonstrate that pharmacological targeting of CD206^+^ macrophages with the RP-182 peptide significantly ameliorates UUO-induced renal fibrosis and tissue damage.

**FIGURE 5:**
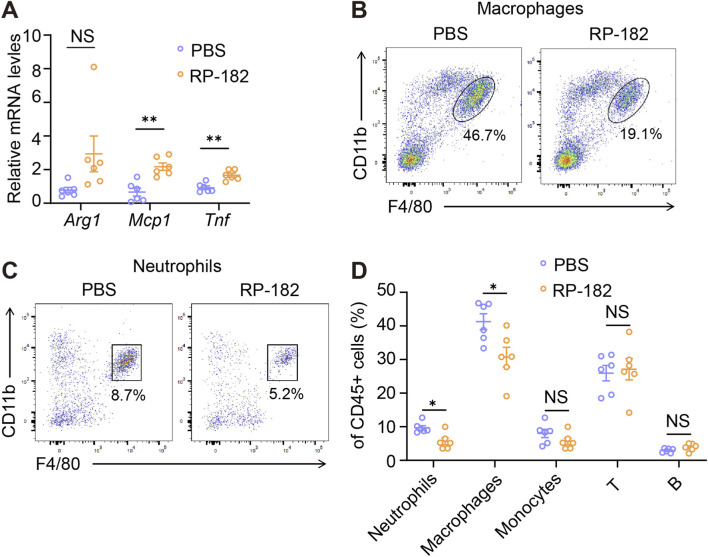
RP-182 peptide inhibited UUO-induced kidney inflammation. **(A)** qPCR analysis for renal expression of *Arg1*, *Mcp1*, and *Tnf* in PBS- and RP-182-treated UUO mice. Representative flow cytometry plots of renal macrophages **(B)** and neutrophils **(C)** in PBS- and RP-182-treated UUO mice. **(D)** Quantification of renal neutrophils, macrophages, monocytes, T cells, and B cells in PBS- and RP-182-treated UUO mice. The results represent mean ± SEM. *p < 0.05, **p < 0.01, NS no significance.

We next investigated whether RP-182 modulates the inflammatory response in obstructed kidneys. Interestingly, transcript levels of the inflammatory mediators *Mcp1* and *Tnf* were elevated upon RP-182 treatment (Figure 5A). Despite this increase in cytokine expression, flow cytometric analysis showed a significant reduction in the infiltration of macrophages and neutrophils in RP-182-treated mice compared to PBS controls (Figures 5B–D). In contrast, the abundances of monocytes, T cells, and B cells were not significantly different between the two groups (Figure 5D). These results suggest that treatment with RP-182 attenuates renal inflammation by limiting the accumulation of key pro-inflammatory leukocytes, including total macrophages.

### 3.4 The RP-182 peptide reversed M2 macrophage polarization but exhibited minimal effect on M1 macrophages

**FIGURE 6:**
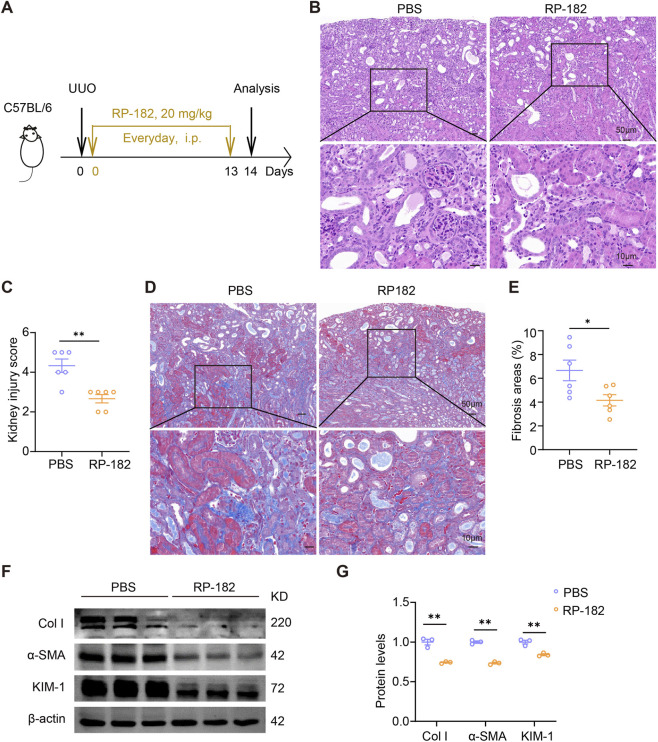
RP-182 peptide inhibited M2 macrophage polarization. **(A)** The schematic of the experimental design. **(B–E)** qPCR analysis for *Cd206*, *Ym1*, *Fizz1*, and *Arg1* expression in BMDMs treated with PBS, IL-4/13, or IL-4/13 + RP-182. N = 6. **(F)** The schematic of the experimental design. **(G)** qPCR analysis for *Il1b*, *Tnf*, and *Nos2* expression in BMDMs treated with LPS or LPS + RP-182. The results represent mean ± SEM. ***p < 0.001, NS no significance.

To investigate the mechanism by which RP-182 modulates macrophage polarization, we isolated bone marrow cells and generated bone marrow-derived macrophages (BMDMs). These cells were stimulated with IL-4 and IL-13 to induce M2 polarization in the presence or absence of the RP-182 peptide (Figure 6A). As expected, IL-4/IL-13 stimulation significantly upregulated the expression of CD206 and other canonical M2 marker genes, including *Ym1*, *Fizz1*, and *Arg1* (Figures 6B–E). Notably, co-treatment with RP-182 markedly suppressed the upregulation of these M2-associated genes (Figures 6B–E). To evaluate the specificity of this effect, we polarized BMDMs toward an M1 phenotype using lipopolysaccharide (LPS) (Figure 6F). In contrast to its pronounced impact on M2 polarization, RP-182 treatment did not significantly alter the expression of key M1 marker genes, including *Il1b*, *Tnf*, and *Nos2* (Figure 6G). Taken together, these results demonstrate that the CD206-targeting peptide RP-182 selectively reverses M2 macrophage polarization without exerting substantial effects on M1 macrophages. The observed decrease in total macrophage infiltration *in vivo* aligns with a specific impact on the recruitment or survival of the profibrotic CD206^+^ subset.

### 3.5 The RP-182 peptide inhibited macrophage-myofibroblast transition (MMT) in M2 macrophages

**FIGURE 7:**
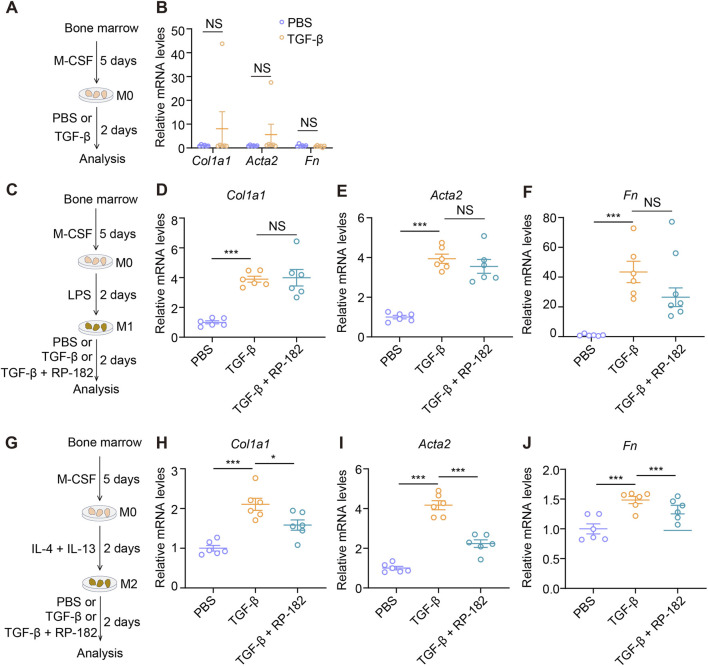
RP-182 peptide inhibited the MMT process. **(A)** The schematic of the experimental design. **(B)** qPCR analysis for *Col1a1*, *Acta2*, and *Fn* expression in M0-BMDMs treated with PBS or TGF-β. N = 6. **(C)** The schematic of the experimental design. **(D–F)** qPCR analysis for *Col1a1*, *Acta2*, and *Fn* expression in M1-BMDMs treated with PBS, TGF-β, or TGF-β + RP-182 peptide. N = 6. **(G)** The schematic of the experimental design. **(H–J)** qPCR analysis for *Col1a1*, *Acta2*, and *Fn* expression in M2-BMDMs treated with PBS, TGF-β, or TGF-β + RP-182. N = 6. The results represent mean ± SEM. *p < 0.05, ***p < 0.001, NS no significance.

Given the critical role of MMT in the progression of renal fibrosis, we next sought to determine whether CD206 targeting modulates this process in different macrophage subtypes, namely, undifferentiated (M0), M1, and M2 macrophages. Interestingly, TGF-β treatment failed to induce the expression of canonical MMT markers, including *Col1a1*, *Acta2*, and *Fn*, in M0 macrophages (Figures 7A,B), suggesting their intrinsic resistance to TGF-β-driven MMT under *in vitro* conditions. In contrast, both M1 and M2 macrophages exhibited a pronounced response to TGF-β, showing significant upregulation of *Col1a1*, *Acta2*, and *Fn* transcripts (Figures 7C–J). Notably, while administration of the RP-182 peptide did not significantly alter TGF-β-induced MMT marker expression in M1 macrophages (Figures 7C–F), it markedly suppressed the upregulation of *Col1a1*, *Acta2*, and *Fn* in TGF-β-stimulated M2 macrophages (Figures 7G–J). Collectively, these results demonstrate that RP-182, by targeting CD206, selectively suppresses TGF-β-induced MMT in M2 macrophages. This subtype-specific inhibition underscores a precise mechanism for its anti-fibrotic action, which is mechanistically distinct from effects on other sources of myofibroblasts.

To establish whether β-catenin inhibition is causally involved in the anti-fibrotic effects of RP-182, we treated IL-4/13-polarized M2 BMDMs with the specific β-catenin pathway inhibitor XAV-939. Notably, the inhibition of β-catenin was sufficient to replicate the effects of RP-182, resulting in a significant reduction in M2 markers (*Ym1*, *Fizz1*, and *Arg1*) (Supplementary Figure S1B–D) and TGF-β-induced MMT markers (*Col1a1*, *Acta2*, and *Fn*) (Supplementary Figure S1F–H). Additionally, simultaneous administration of RP-182 and XAV-939 did not have an additive inhibitory effect, indicating a shared mechanism of action. In summary, these findings establish that RP-182 impedes M2 macrophage polarization and MMT by suppressing the β-catenin signaling pathway.

### 3.6 Targeting CD206 by the RP-182 peptide inhibited β-catenin signaling pathway

**FIGURE 8:**
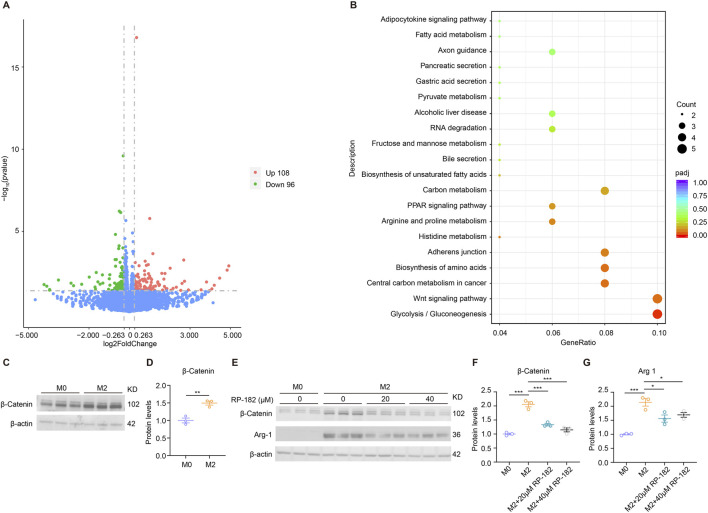
RP-182 peptide inhibited Wnt/β-catenin signaling pathway in M2 macrophages. **(A)** Volcano plot showing differentially expressed genes between M2-BMDMs treated with RP-182 versus PBS. **(B)** Kyoto encyclopedia of genes and genomes (KEGG) enrichment analysis of the upregulated genes in RP-182-treated M2-BMDMs. The top 20 pathways were shown. **(C)** Immunoblots and **(D)** quantification for β-catenin expression in M0-and M2-BMDMs. N = 3. **(E)** Immunoblots and **(F,G)** quantification for β-catenin and Arg-1 expression in M0-and M2-BMDMs treated with 0, 20, or 40 μM RP-182 peptide. N = 3. The results represent mean ± SEM. *p < 0.05, **p < 0.01, ***p < 0.001.

To elucidate the mechanisms by which RP-182 regulates M2 macrophage polarization, we conducted bulk RNA sequencing (RNA-seq) on IL-4/IL-13-treated BMDMs with or without exposure to the RP-182 peptide. Our analysis identified 108 significantly upregulated and 96 downregulated genes (fold change >1.2, p < 0.05) (Figure 8A). Gene set enrichment analysis revealed that the downregulated genes were primarily associated with pathways such as glycolysis/gluconeogenesis, amino acid biosynthesis, and Wnt signaling (Figure 8B). Mounting evidence has demonstrated that the Wnt/β-catenin signaling pathway plays a critical role in regulating M2 macrophage polarization. To investigate whether the antifibrotic effects of RP-182 involve modulation of this pathway, we assessed its impact on β-catenin activation in polarized macrophages. Consistent with previous reports, M2 macrophages exhibited significantly elevated β-catenin protein levels compared to undifferentiated M0 macrophages (Figures 8C,D), reinforcing the association between β-catenin signaling and the M2 phenotype. Notably, treatment with the RP-182 peptide significantly reduced β-catenin expression in M2 macrophages (Figures 8E,F). This downregulation was accompanied by a marked decrease in the expression of Arg1 (Figure 8G), a canonical M2 marker that is known to be transcriptionally regulated by β-catenin. Together with the observed suppression of M2-associated genes (Figures 6B–E), this establishes that RP-182 inhibits the M2 transcriptional program and decreases key effector proteins, including Arg1, by attenuating β-catenin signaling. These findings suggest that RP-182 inhibits M2 macrophage polarization, at least in part, through suppression of the β-catenin signaling pathway.

## 4 Discussion

In this study, we provide compelling evidence that chronic ureteral obstruction leads to substantial accumulation of CD206^+^ macrophages in UPJO patients and UUO murine model. Importantly, RP-182 treatment not only reduced fibrosis and tubular injury but also specifically suppressed M2 macrophage polarization and MMT process through a mechanism involving inhibition of the β-catenin signaling pathway. These findings position CD206 as a promising therapeutic target and RP-182 as a potential candidate for antifibrotic therapy.

Macrophages are vital players in organ fibrosis (Jiang et al., 2024). Recent advances in macrophage-targeted therapies have unveiled promising strategies for mitigating organ fibrosis, with particular progress in renal fibrosis (Hou et al., 2024; Zhang J. et al., 2025). Key mechanisms involve modulating macrophage polarization, metabolic reprogramming, and specific profibrotic pathways (Jiang et al., 2024). For instance, in renal fibrosis, suppression of the RNA demethylase ALKBH5 in macrophages reduces M2a polarization and downregulates TGF-β1, Arg1, and CD206 expression, thereby alleviating fibrotic progression via its downstream target Retnla (Zheng et al., 2025). Similarly, inhibition of the voltage-gated potassium channel Kv1.3 with Margatoxin (MgTx) shifts macrophage polarization from pro-inflammatory M1 to anti-inflammatory M2 phenotypes, attenuating renal inflammation and fibrosis through suppression of the ERK/NF-κB pathway (Chen et al., 2025b; Li et al., 2025). Additionally, macrophage-derived Galectin-3 exacerbates renal fibrosis by stabilizing TGF-β receptors and pro-TGFβ1, and its genetic or pharmacological inhibition significantly improves outcomes in diabetic kidney disease models (Chen et al., 2025a). A recent study identifies the major profibrotic macrophage subset (Fn1^+^ Spp1^+^ Arg1^+^) in the kidney and then constructs a glycopeptide called bioactivated *in vivo* assembly PK (BIVA-PK), which is able to deplete Fn1^+^ Spp1^+^ Arg1^+^ macrophages (Ouyang et al., 2024). BIVA-PK specifically binds to and is internalized by profibrotic macrophages, leading to macrophage cell death, reshaping the renal microenvironment, and suppressing profibrotic immune responses (Ouyang et al., 2024).

Beyond the kidney, strategies targeting macrophage-specific metabolic pathways and engineered therapies show broad antifibrotic potential. In pulmonary fibrosis, Mefunidone targets succinate dehydrogenase (SDH) in macrophages, inhibiting succinate accumulation and suppressing the SDH-GPR91 axis to block the transformation of MMP12^+^ CCL2^+^ profibrotic macrophages and their crosstalk with fibroblasts (Zhang X. et al., 2025). For cardiac fibrosis, chimeric antigen receptor macrophages (CAR-M) designed to target fibroblast activation protein effectively phagocytose activated fibroblasts, reducing collagen deposition and improving cardiac function in preclinical models without significant toxicity (Gao et al., 2024). These approaches highlight the versatility of targeting macrophage subsets through metabolic regulation, surface markers, or engineered receptors. However, challenges remain in achieving cell-type specificity, ensuring long-term safety, and translating these findings into clinical applications. Future efforts should focus on approaches that integrate macrophage-specific targeting with antifibrotic agents to address the complexity of fibrotic microenvironments across organs.

The prominent infiltration of CD206^+^ macrophages in human UPJO tissues and murine UUO kidneys underscores their critical role in fibrotic progression. Their spatial association with α-SMA^+^ cells and strong correlation with ECM components suggests that these cells might contribute directly to myofibroblast activation and matrix accumulation. This aligns with established literature indicating that M2 macrophages secrete profibrotic mediators such as TGF-β and PDGF, thereby fostering a microenvironment conducive to tissue scarring (Shinoda et al., 2023; Jiang et al., 2024). Interestingly, another CD206-targeting HDP, RP-832c, has also been reported to be able to induce a conformation change and apoptosis in CD206-positive M2 macrophages and repolarization toward the M1 phenotype (Jaynes et al., 2020). Notably, RP-832c exhibits a similar reduction in lung fibrosis compared to the FDA-approved drugs Pirfenidone and Nintedanib (Ghebremedhin et al., 2023). Pirfenidone, a broad-spectrum antifibrotic agent, acts by modulating TGF-β signaling, reducing inflammation, and scavenging reactive oxygen species, showing clinical benefit in idiopathic pulmonary fibrosis (King et al., 2014). However, its systemic use is often hindered by side effects like gastrointestinal upset and photosensitivity, and its mechanism lacks specificity towards profibrotic immune cells. In contrast, RP-182 offers a targeted immunomodulatory approach that selectively reprograms CD206^+^ macrophages and inhibits the β-catenin pathway downstream, potentially providing a more precise intervention with reduced risk of off-target effects. Although direct preclinical comparisons in renal fibrosis are necessary for future studies, our findings indicate that RP-182 significantly reduces collagen deposition and injury markers through this unique, macrophage-targeted mechanism, underscoring its potential as an innovative and potentially more specific therapeutic candidate.

Our findings demonstrate that the CD206-targeting peptide RP-182 effectively suppresses M2 macrophage polarization and MMT by inhibiting the β-catenin signaling pathway. This aligns with the emerging recognition of β-catenin as a pivotal regulator of macrophage functional polarization (Sarode et al., 2020; Liu et al., 2022). The use of a β-catenin inhibitor in our functional perturbation experiment unequivocally shows that inhibition of this pathway alone is adequate to replicate the anti-fibrotic reprogramming impacts of RP-182 on M2 macrophages. This direct evidence substantially reinforces the assertion that β-catenin plays a pivotal role as a downstream mediator of RP-182s mechanism of action. Recent investigations have consistently shown that activation of the Wnt/β-catenin pathway drives macrophages toward a profibrotic M2 phenotype (Cui et al., 2024; Deng et al., 2024) and facilitates MMT, processes integral to fibrogenesis in various organs (Yang et al., 2019; Shen S. et al., 2025). For instance, in lung fibrosis, inhibition of β-catenin signaling via compounds like Icariside II was shown to effectively blunt the M2 macrophage program and ameliorate fibrotic pathology (Deng et al., 2024). Furthermore, environmental profibrotic stimuli such as PFDA (perfluorodecanoic acid) promote cancer metastasis by enhancing macrophage M2 polarization through a Wnt/β-catenin-dependent mechanism (Cui et al., 2024).

The significance of MMT as a mechanism in fibrotic diseases has gained increasing recognition (Tang et al., 2018; Shen S. et al., 2025; Xia et al., 2025). Myofibroblasts, the primary matrix-producing cells in fibrosis, originate from various sources, including resident fibroblasts, epithelial cells, endothelial cells, and importantly, macrophages (via MMT) (Kuppe et al., 2021). In chronic kidney disease, MMT contributes significantly to the myofibroblast population (Zhang et al., 2024). Recent studies have demonstrated that MMT is driven by key signaling pathways, notably the TGF-β/Smad axis and Syk/NF-κB cascades (Vierhout et al., 2021; Shen L. et al., 2025). Our study identifies a novel function of RP-182: the selective inhibition of TGF-β-induced MMT exclusively in M2 macrophages, sparing their M1 counterparts. This cellular specificity is consistent with its mechanism of action via CD206, a receptor predominantly expressed on M2 macrophages. Therefore, the primary anti-fibrotic effect of RP-182 observed *in vivo* is likely mediated through the reprogramming of this key profibrotic immune population, rather than through direct effects on resident fibroblasts or epithelial cells.

The potential mechanistic link between RP-182 engagement of CD206 and subsequent β-catenin inhibition, as suggested by our data, could involve several interconnected processes and merits further investigation. Binding of RP-182 to CD206 may induce conformational changes in the receptor and modulate its endocytic recycling or intracellular signaling, potentially disrupting downstream pathways that converge on β-catenin stability or transcriptional activity (Maurice and Angers, 2025). Given that β-catenin is regulated by diverse upstream signals (Yan et al., 2025), it is possible that CD206 engagement by RP-182 initiates intracellular events that ultimately suppress β-catenin signaling; however, the precise molecular mechanisms underlying this association remain unclear and warrant further elucidation.

While our findings demonstrate the therapeutic potential of RP-182, several translational considerations merit discussion. First, regarding administration, the intraperitoneal delivery used here is a standard pre-clinical proof-of-concept route; future development must explore more feasible chronic administration strategies, such as subcutaneous injection or the engineering of stabilized peptide analogues. Second, comprehensive safety and pharmacokinetic profiles are required. Although no overt toxicity was observed in our acute model, dedicated long-term studies in chronic disease settings are indispensable to fully assess potential off-target effects and monitor detailed renal and systemic function. Finally, to establish broader relevance, the efficacy of RP-182 should be evaluated across the etiological spectrum of chronic kidney disease, including diabetic and hypertensive nephropathy. These steps are crucial to bridge the gap between our promising mechanistic findings and future clinical development.

## 5 Conclusion

In conclusion, our findings demonstrate that targeting CD206^+^ macrophages with RP-182 attenuates renal fibrosis by inhibiting both M2 polarization and MMT, supporting its development as a novel therapeutic agent for obstruction-induced renal fibrosis.

## Data Availability

The datasets presented in this study can be found in online repositories. The names of the repository/repositories and accession number(s) can be found below: https://ngdc.cncb.ac.cn/omix, OMIX012327.

## Supplementary material

SUPPLEMENTARY FIGURE S1 XAV-939 and RP-182 inhibit the polarization of M2 macrophages and the process of MMT. **(A)** The schematic of the experimental design. **(B–D)** qPCR analysis for *Ym1*, *Fizz1*, and *Arg1* expression in BMDMs treated with IL-4/13, IL-4/13 + XAV-939, IL-4/13 + RP-182, or IL-4/13 + RP-182 + XAV-939. N = 6. **(E)** The schematic of the experimental design. **(F–H)** qPCR analysis for *Col1a1*, *Acta2*, and *Fn* expression in M2-BMDMs treated with TGF-β, TGF-β + XAV-939, TGF-β + RP-182, or TGF-β + RP-182 + XAV-939. N = 6. The results represent mean ± SEM. *p < 0.05, ***p < 0.001, NS no significance.
