# Supplementary figures and images for: RP-182 alleviated obstruction-induced renal fibrosis by reprogramming CD206+ macrophages
Source: Front Pharmacol. 2026 Jan 30;17:1739457. doi: 10.3389/fphar.2026.1739457 (PMC12900717; doi:10.3389/fphar.2026.1739457)

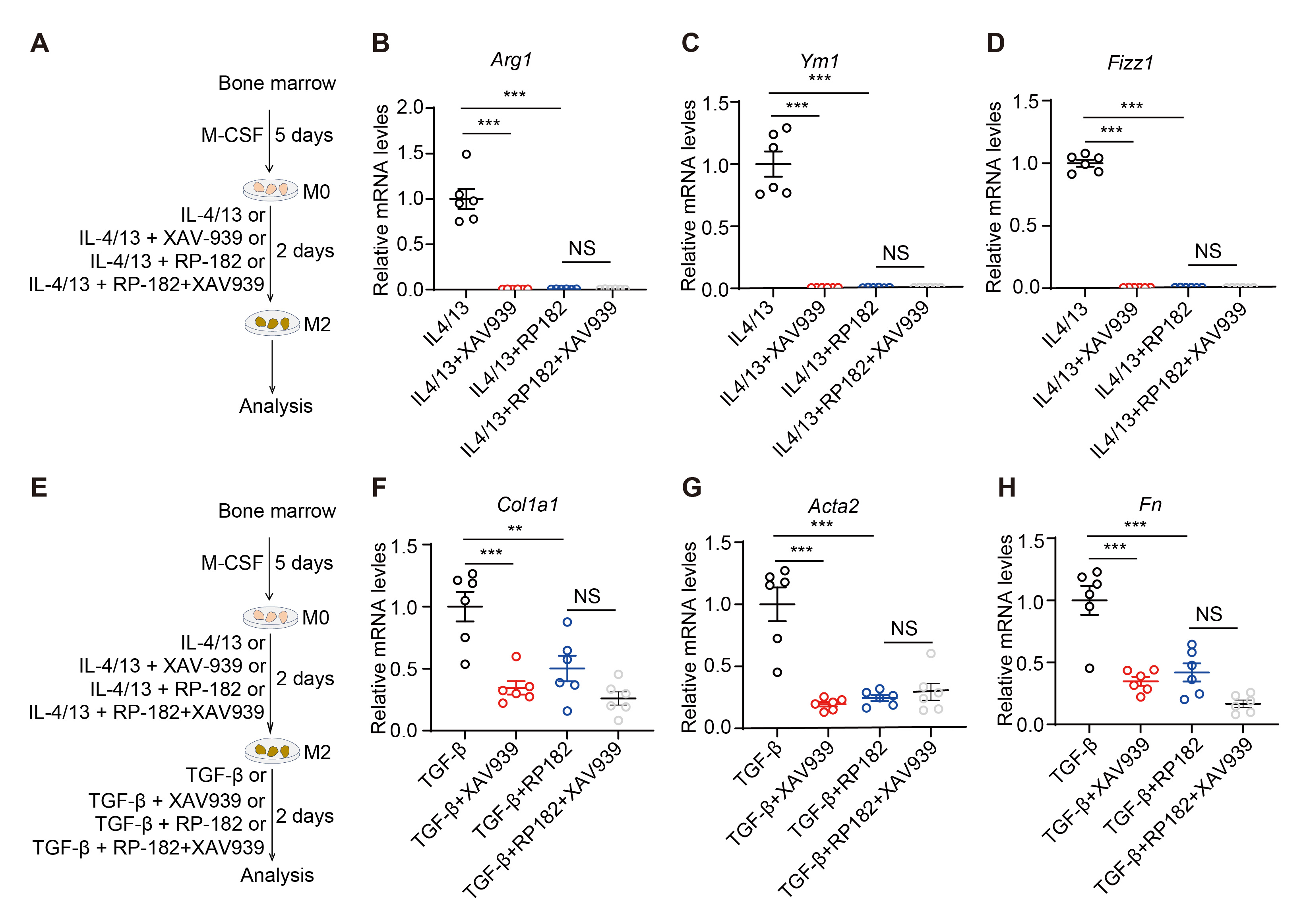

Supplement: Supplementary file 1 [file Image1.jpeg]
